# An investigation of factors associated with psychiatric hospital admission despite the presence of crisis resolution teams

**DOI:** 10.1186/1471-244X-7-52

**Published:** 2007-10-02

**Authors:** Mary-Anne Cotton, Sonia Johnson, Jonathan Bindman, Andrew Sandor, Ian R White, Graham Thornicroft, Fiona Nolan, Stephen Pilling, John Hoult, Nigel McKenzie, Paul Bebbington

**Affiliations:** 1 Department of Mental Health Sciences, Royal Free and University College Medical Schools, UCL, London, UK; 2 Health Services Research Department, Institute of Psychiatry, Kings College London, London, UK; 3 Central and North West London Mental Health Trust, London, UK; 4 MRC Biostatistics Unit, Cambridge, UK; 5 Camden and Islington Mental Health and Social Care Trust, London, UK; 6 CORE, Department of Clinical Health Psychology, UCL, London, UK

## Abstract

**Background:**

Crisis resolution teams (CRTs) provide a community alternative to psychiatric hospital admission for patients presenting in crisis. Little is known about the characteristics of patients admitted despite the availability of such teams.

**Methods:**

Data were drawn from three investigations of the outcomes of CRTs in inner London. A literature review was used to identify candidate explanatory variables that may be associated with admission despite the availability of intensive home treatment. The main outcome variable was admission to hospital within 8 weeks of the initial crisis. Associations between this outcome and the candidate explanatory variables were tested using first univariate and then multivariate analysis.

**Results:**

Patients who were uncooperative with initial assessment (OR 10.25 95% CI-4.20–24.97), at risk of self-neglect (OR 2.93 1.42–6.05), had a history of compulsory admission (OR 2.64 1.07–6.55), assessed outside usual office hours (OR 2.34 1.11–4.94) and/or were assessed in hospital casualty departments (OR 3.12 1.55–6.26), were more likely to be admitted. Other than age, no socio-demographic features or diagnostic variables were significantly associated with risk of admission.

**Conclusion:**

With the introduction of CRTs, inpatient wards face a significant challenge, as patients who cooperate little with treatment, neglect themselves, or have previously been compulsorily detained are especially likely to be admitted. The increased risk of admission associated with casualty department assessment may be remediable.

## Background

Crisis resolution teams offer an alternative to hospital admission in mental health crises. They target people who would be admitted acutely to hospital without their intervention, providing intensive home treatment whenever feasible with 24 hour availability, daily or twice daily home visits, control over access to in-patient beds and a range of interventions focused both on symptoms and immediate social problems. Nationwide introduction of crisis resolution teams is a requirement in England [1], and the National Service Mapping for Mental Health in 2005 [2] indicated that 267 CRTs had been established. Similar teams were established throughout the state of Victoria, Australia in the 1990s [3], and diversion from acute admission through the provision of a rapid response in the community and frequent multidisciplinary home visits has been the aim of a variety of model services introduced in several countries over the past few decades. [4-6].

Recent evidence suggests that intensive home treatment delivered by CRTs does reduce admission rates. Two studies, one naturalistic and one a randomised controlled trial, have investigated outcomes of CRT care in Islington, London [7,8]. Both showed a significant reduction in hospital admissions at 8 weeks and 6 weeks respectively for those given access to a CRT. An analysis of the impact nationally of CRT introduction suggests a significant reduction in admission rates associated with this, especially for CRTs with a higher degree of model fidelity [9]. These recent findings cohere with older studies from various countries involving the evaluation of teams offering acute home treatment, even though the characteristics of the teams offering home treatment and the wider service context are substantially different. [10,11].

While intensive home treatment teams appear to have some impact on admissions, all studies indicate that a substantial group of patients is admitted to hospital despite the availability of such teams. As yet, little is known about the factors associated with patients being admitted to hospital despite the availability of such an alternative. A better understanding of this may be helpful in several ways. Firstly, such evidence may help inform further development of intensive home treatment services as there may be scope for modifying them so they are better able to meet the needs of those currently admitted. Secondly, the composition of in-patient populations may change as a result of the introduction of home treatment, and service planning and provision in hospitals should target the groups most likely to be admitted. Thirdly, clinicians deciding whether to admit patients may find it helpful to know which groups are most likely to be successfully managed at home. And finally, other alternatives to hospital admission such as residential crisis services might meet the needs of some of those currently admitted despite the availability of CRTs.

A few previous studies have investigated variables associated with hospital admission in settings where a community alternative is available [12-22]. Table 1 lists them. Most have substantial limitations. Firstly, only three relate to alternatives that are purely short-term acute home treatment teams – most are hybrid services delivering both acute and continuing care [12-14]. Secondly, almost half the papers did not involve a multivariate analysis, so that factors independently associated with admission when others are adjusted for could not be identified [12,13,15-17]. Thirdly four papers had samples of fewer than 100 subjects [[12,13,15], and [18]]. Finally, the candidate variables investigated differed considerably from one study to another, with none investigating anything close to a full set of candidate variables. Police referral, psychosis and being suicidal were the most frequent observed positive findings.

**Table 1:** Literature investigating factors associated with admission to psychiatric hospital in the context of an alternative to hospital

**Paper and country of origin**	**Comparison groups and statistical analysis**	**Description of the service alternative to hospital admission**	**Variables found to be significantly associated with psychiatric hospital admission**
Brimblecombe N 1999 [17] Brimblecombe N 2003 [19] UK	197 subjects in HT compared to 121 admitted to hospital Chi Squared test. 231 subjects accepted for HT compared to 62 subjects requiring hospital admission. Forward logistic regression analysis	Typical crisis resolution team however only provided 12-hour service daily including weekends.	Hypo manic presentation Personality disorder High suicidal ideation (p < 0.01) Previous hospital admission (p < 0.01)
Dean C1990 [12] UK	65 subjects treated by HT compared to 34 subjects admitted to hospital. Chi-squared test.	Typical crisis resolution team.	Assessment outside office hours. Assessment in hospital or police station as opposed to home or outpatients Living alone. Not married. Younger (men). Previous admissions. Previous compulsory admissions. Violent during episode of illness.
Harrison J 2001 [14] UK	101 accepted onto HT compared to 94 refused HT. Forward stepwise logistic regression analysis	Twenty-four hour service with treatment offered either in patients own home or at the team base. Hybrid between day hospital and home treatment. Likelihood of being accepted to home treatment main outcome.	Diagnosis of less severe disorder (not Schizophrenia-spectrum or severe mood disorder) less likely to be accepted to home treatment. Location of referral not in community or outpatients less likely to be accepted. Out of 9 am–5 pm hours referrals less likely to be accepted. Referral from less senior practitioner less likely to be accepted.
Bracken P 1999 [13] UK	53 patients admitted to HT versus 63 admitted to hospital. Chi-squared test.	Typical crisis resolution team. Decision to admit to hospital or home treatment team made by sector or on-call consultant.	Not on CPA. Less likely to have a severe mental illness such as schizophrenia and manic depression. Primary diagnosis of personality disorder. Primary diagnosis of drug/alcohol problems.
Abas M 2003 [17] New Zealand	Reasons for admission and alternatives to admission were rated for a consecutive sample of 255 admissions to an acute psychiatric unit. Descriptive analysis only	Alternative care package included residential facilities with different levels of support, or home visits from a mental health nurse at least once a day. 'Crisis Team' gate keep all admissions. No further information is given however on the intervention provided by the crisis team.	Functional psychosis and marked social deprivation. Reasons cited for admission: reinstatement of medication, intensive observation, risk to self and risk to others.
Guo S 2001 [20] USA	Matched case-control study of 4,106 subjects who had hospital based intervention compared to 1,696 subjects that had crisis intervention. Cox proportion hazards model.	Community-based mobile crisis program provided by a multidisciplinary team including crisis intervention specialists, registered nurses and psychiatrists. The team would review a case, attempt to stabilize the crisis recommend appropriate services and provide follow-up. ? Round the clock cover.	Referred by legal system Referred by psychiatric hospital or other treatment facility Primary diagnosis of schizophrenia, affective psychosis or other psychosis. Primary presenting problem being a suicidal gesture. Not with a primary diagnosis of drugs or alcohol dependency. Homeless Unemployed
Schnyder U 1999 [21] Switzerland	Of 3611 psychiatric emergencies 1093 cases offered no further intervention were compared to 1287 cases offered outpatient crisis intervention and 1231 cases admitted to hospital. Chi-squared followed by logistic regression analysis	Outpatient crisis intervention offered but little more information is given about what this comprises.	Referral by police or by health professionals Diagnosis of psychotic disorder History of previous hospitalization Other factors: Male Single or divorced Living alone Less skilled worker/unemployed Less likely to self refer More previous hospital admissions More severe conditions
Slagg NB 1983 [18] USA	Characteristics of three dispositional groups of 50 randomly selected subjects each were compared Multivariate analysis and validated in a second sample	Outpatient crisis program, which offers 6 visits, initiated within 24 hours of evaluation and program attempts to link patients to continuing treatment services if appropriate.	More psychologically impaired Psychotic Unlikely to self-refer Educated Unemployed Expressing acting out behaviour
Segal S 1996 [22] USA	Interviewed? non-psychiatric Clinicians regarding their disposition decisions of 425 patients attending psychiatric emergency services. Multivariate analysis	Less restrictive alternative included supervised residential placement, including a placement with willing and responsible relative, crisis housing, halfway houses, board and care homes and foster family care.	Less engagement/cooperation with clinician Referral by police
Walsh SF 1986 [15] USA	Compared 30 Emergency housing project (EHP) failures with 30 who were maintained and treated at the EHP Bi-variate discriminate function analysis	The emergency housing project is a short-term transitional residential setting designed to enable acutely ill psychiatric patients to be treated in the community as outpatients. Housed in a single room occupancy hotel staffed 24 hours a day by mental health workers supervised by a clinical social worker. The goals include psychological and social stabilization of the patient. Median length of stay is 11.2 days. Staff does not supervise medication.	Use of illicit substances Non-compliance with medication Uncooperatively with agencies

HT-Home Treatment

Many other investigations have been published of factors which may influence the decision to admit from the casualty department or emergency room [23], but these have not been included here as no innovative alternative to hospital admission was available.

### Aims

The aim of this study was to explore which of a comprehensive set of explanatory variables were independently associated with hospital admission within 8 weeks of an initial crisis presentation in a large cohort of patients with access to a CRT. Secondary objectives were to explore variables associated with admission within 6 months, with total bed days at 8 weeks and 6 months and with compulsory hospital detention.

## Methods

The data used were collected whilst conducting 3 studies in deprived London Boroughs comparing outcomes of CRTs with standard care. Two are published [7,8]; the third (in North Southwark) used a methodology closely based on that employed by Johnson et al. [8]. Only data from the experimental groups are included in the analyses for the current study, so that all in the sample on which these analyses are based had access to CRT care.

### Setting and description of services

Three different CRTs were investigated in the three studies whose results have been pooled for this analysis. Two were located in the London Borough of Islington and one in the London Borough of North Southwark. These are all deprived inner London boroughs with ethnically very mixed populations. The Islington sectors were served by well established community mental health teams (CMHTs) and a liaison team comprising of psychiatric nurses, a junior doctor and access to a consultant psychiatrist. The liaison team assesses any patient who attends the casualty department and is deemed to need mental health input. They operate between the hours of 8.00 am – 10.00 pm in the casualty department of the local general hospital. Two crisis houses, one women-only, were available as alternatives to hospital admission and were operating prior to the introduction of CRTs. The North Southwark sector was also served by a CMHT and out of hours liaison services at two local general hospitals. In addition there was an early intervention service for psychosis for the whole borough operating between 9.00 am and 5.00 pm. All the CRTs were multidisciplinary. The two Islington teams had junior doctors within the teams and senior medical input from local CMHT sector psychiatrists. The North Southwark service had a dedicated CRT consultant psychiatrist and a junior doctor on rotation from the local psychiatric training scheme within the team. All three teams provided 24 hour cover with a gate-keeping role, so that any patient assessed as requiring hospital admission could only be admitted if the CRT had agreed that home treatment was not feasible. The teams monitored symptoms, administered medication where necessary, identified and offered advice and help with social stresses that might be contributing to the crisis, and made appropriate follow-up arrangements with other local services once the crisis was resolving and discharge approaching. Patients could be seen twice daily if needed and were able to contact the CRT at any time. The CRTs arranged hospital admission if their intervention proved to be unsuccessful or the patient was deemed unsuitable for home treatment. The model implemented by all the teams essentially conformed to guidelines laid out by the Policy Implementation Guide [2]. The operation of CRTs has been described in more detail by Johnson [24].

### Sample

The first Islington study was designed as a natural experiment comparing outcomes of patients presenting in crisis before and after the introduction of the South Islington CRT. Our study only included the group from the second phase of the study, who presented in crisis once the CRT had become available and begun to assess all patients presenting in crisis. No patient who met the criteria for being in crisis, as judged by an expert panel, was excluded [7]. The key features of the definition of crisis were that the person had to show deterioration in mental health or social functioning, and that this deterioration had led to concerns about their safety or the safety of others which were sufficiently great for an immediate change in clinical management to be required. The cohort of 123 patients used included in the current study was recruited over a 9 month period following the introduction of CRTs. Assessments were carried out on those identified as being in crisis immediately after identification of crisis, 6 weeks and 6 months afterwards.

The second Islington study from which patients have been drawn for the current analysis was the North Islington study [8], which used a randomised controlled trial design. Patients were eligible for the trial if they were resident in the study area and presented in a crisis severe enough for clinicians to judge that hospital admission was warranted. Further requirements were that *either *they had decision making capacity at the time of the crisis and consented to randomisation *or *that they lacked capacity, but had previously received information about the study and had not chosen to opt out in advance (1160 service users were contacted in advance, of whom exactly 100 opted out) *or *that they lacked capacity and had not already been informed about the study, but had a carer who was willing to give initial assent to their inclusion. Two hundred and sixty people were randomised during the recruitment period, while 104 were admitted to hospital without entering the study. The sample included in the analyses for the current paper consisted of the 135 participants who were randomised to the experimental group and whose care thus involved assessment by the CRT and intensive home treatment whenever feasible. This methodology was replicated in North Southwark where 100 patients were recruited to the experimental group. All three studies received local research ethics committee approval. In both studies those patients who had access to CRTs had significantly reduced admission rates compared to those who did not have access. For the South Islington study [7] admission rates at 6 weeks were reduced from 70% to 48% with a p-value of 0.002. In the randomised controlled study [8] hospital admission at 8 weeks was again significantly reduced in the experimental group with access to CRT (OR 0.19 95% CI   0.11–0.32). Neither studies showed any impact of CRT access on patients being admitted to hospital compulsorily.

### Data collection: explanatory variables

Table 2 shows the baseline variables used for this study. These were obtained from the best available data source: patients supplied socio-demographic and clinical information when feasible, but this was supplemented by staff reports and case notes where interview information could not be obtained. The main presenting symptoms were identified and rated by staff, as was cooperativeness at time of assessment. Ratings of the severity of clinical and social problems were collected using the Health of the Nation Outcomes Scales (HoNOS, [25]). Current risk of self harm, violence, self-neglect with serious lack of caution or vulnerability from exploitation from others was evaluated using staff ratings on the Threshold Assessment Grid (TAG) [26] for the North Islington and North Southwark studies. In the South Islington study a set of structured questions on severity of risk, eliciting ratings on a five point scale encompassing no risk, mild risk, moderate risk and severe risk was used. For the purposes of this analysis, a rating of moderate risk on the scale used in South Islington was treated as equivalent to a rating of moderate risk on the TAG.

**Table 2:** Univariate associations between candidate variables and admission by eight weeks

**Variable (N = for this analysis)**	**Percentage within each category admitted at 8 weeks:- No. admitted/total number in category (%)**	**Odds ratio for being admitted at 8 weeks, (95% confidence interval)**	**p-value**
**Overall rate of admission to hospital within 8 weeks (358)**	157/358 (44%)		
**Sex: male (358)**	94/185 (51%)	1.80(1.18 to 2.75)	0.006
**Ethnic Group: (358)**			
***White European***	107/246 (44%)	Reference group	0.07
***Black Caribbean***	11/29 (38%)	0.79 (0.36 to 1.75)	
***Black African***	26/42 (62%)	2.11 (1.08 to 4.13)	
***Black Other***	5/11 (46%)	1.08 (0.32 to 3.64)	
***Asian***	6/17 (35%)	0.71 (0.25 to 1.98)	
***Mixed or other***	2/13 (15%)	0.24 (0.05 to 1.09)	
**Homeless including temporary accommodation (353)**	17/32 (53%)	1.52 (0.73 to 3.15)	0.26
**Living alone, including with children under 18 years but no adults (357)**	86/203 (42%)	0.88 (0.58 to 1.34)	0.56
**Employed (including voluntary or sheltered employment, studying) (358)**	36/74 (49%)	1.28 (0.76 to 2.13)	0.35
**Comorbid substance misuse (345)**	62/141 (44%)	1.08 (0.70 to 1.66)	0.74
**Psychiatric admission in past two years (355)**	60/115 (52%)	1.67 (1.06 to 2.61)	0.03
**History of being compulsorily admitted (346)**	75/141 (53%)	2.06 (1.33 to 3.18)	0.001
**On CPA (see table 2 footnote?)**	74/176 (42%)	0.85 (0.56 to 1.29)	0.44
**Moderate or severe ratings by staff regarding: (357)**			
***Risk of deliberate self harm***	53/144 (37%)	0.61(0.40 to 0.94)	0.025
***Risk of unintentional self harm ****(e.g. through self neglect)*	59/96 (62%)	2.65 (1.64 to 4.29)	<0.001
***Risk from others ****(e.g. through assault, exploitation by others)*	61/120 (51%)	1.52 (0.98 to 2.36)	0.06
***Risk of harm to others *Presenting symptoms in crisis (358)**	68/110 (62%)	2.87 (1.81 to 4.57)	<0.001
***Psychotic symptoms***	112/213 (53%)	2.46 (1.58 to 3.84)	<0.001
***Depressive symptoms***	61/172 (36%)	0.52 (0.34 to 0.79)	0.002
***Manic symptoms***	36/67 (54%)	1.63 (0.96 to 2.78)	0.07
**Uncooperative with process of arranging and carrying out initial assessment (350)**	59/76 (78%)	6.98 (3.85 to 12.66)	<0.001
**Violence in two years before crisis (343)**	52/91 (57%)	2.10 (1.30 to 3.41)	0.003
**Deliberate self harm in past two years (351)**	43/121 (36%)	0.63 (0.40 to 1.00)	0.05
**Assessment carried out in Accident and Emergency department (357)**	60/127 (47%)	1.23 (0.79 to 1.90)	0.36
**Assessment carried outside office hours (340)**	66/119 (56%)	2.11 (1.34 to 3.32)	0.001
**Referred by police (358)**	26/39 (67%)	2.87 (1.42 to 5.79)	0.003
**Self-referral (358)**	46/140 (33%)	0.47 (0.30 to 0.73)	0.001
**Primary or secondary diagnosis of personality disorder (348)**	18/46 (39%)	0.83 (0.44 to 1.56)	0.56
**Crisis team available to patient (358):**			
***North Islington team (135)***	29/135 (22%)	reference	<0.001
***South Islington team (123)***	63/123 (51%)	3.84 (2.23 to 6.60)	
***North Southwark team (100)***	65/100 (65%)	6.79 (3.80 to 12.14)	
**Characteristic**	**Mean and standard deviation for whole group**	**Mean difference between those admitted at 8 weeks and those not admitted**	**p-value**
**Mean Age (358)**	38.0(12)	1.88 (-0.63 to 4.38)	0.14
**Severity of clinical and Social Problems:**			
**HoNOS total symptom severity (343)**	5.0(2.0)	-0.11 (-0.54 to 0.31)	0.60
**HoNOS total social problem severity (344)**	6.6(4.6)	-1.84 (-2.81 to -0.88)	0.001

### Outcome measures

The primary outcome measure on which analysis was based was whether the patient was admitted within 8 weeks of the initial crisis assessment. Secondary outcome measures were whether the patient was admitted within 6 months, whether the patient was admitted compulsorily under the 1983 Mental Health Act and bed days in hospital at both 8 weeks and 6 months.

### Analysis

The choice of variables was based on previous literature, as reviewed in the introduction, with 3 further variables (risk of unintentional harm to self, for example through self neglect or incautious behaviour, risk from others and which crisis team was providing the service) chosen on clinical grounds as potentially important but not investigated in previous studies. All the variables used are shown in Table 2. The TAG variables relating to severity were not normally distributed in their initial form and were converted into binary variables indicating whether or not a risk of at least moderate severity was present.

Univariate tests (chi squared tests and t tests) were first used to compare those admitted and those not admitted by 8 weeks on each explanatory variable. Logistic regression was then used to test which variables retained an independent effect after adjustment for other explanatory variables. All analysis used STATA statistical software, version 8 (StatCorp, 2003). Less than 10% of the data were missing, but exclusion of all cases with missing data would nonetheless have resulted in substantial loss of data. To avoid this, we used multiple imputation, which fills in the missing values based on values of other variables and a missing at random assumption [27]. Unlike other methods of imputation, multiple imputation acknowledges uncertainty about the missing values by creating several imputed datasets. Each imputed dataset is analysed separately and the results are combined in a way that correctly allows for uncertainty about the missing values [28]. We generated five imputed datasets using the ice command [29], including all variables in Table 2 in the imputation model. We analysed the imputed data using the micombine command [29]. This logistic regression procedure was repeated for admission by 6 months and compulsory detention, and linear regression was used to analyse bed use.

## Results

The three data sets yielded a total of 379 cases. Twenty-one had missing data for the primary outcome and were therefore dropped from the analysis. This represents just over 5% of the total. Amongst the 358 cases analysed, the number of missing cases per variable ranged from 0 to 18, with no missing data at all for 10 variables. These missing data were imputed as described above. Data are available on timing of admission for 129 of the 157 people who became in-patients during the first 8 weeks of follow-up. Of this group, 90 (70%) were admitted within 24 hours of the assessment, 16 (12%) between 1 and 7 days in the community and 23 (18%) after at least 7 days in the community.

Table 2 shows the results of univariate analyses relating to admission within 8 weeks. It should be noted that no adjustment has been made for multiple testing. A large number of the candidate variables were associated with admission on these analyses. Risk of admission was highly associated with which of three CRTs the patient had access to, with those covered by the North Islington team significantly less likely to be admitted. The highest admission rate (78%) was for patients rated by staff as uncooperative with the process of arranging and carrying out the initial assessment. People with psychotic symptoms as a presenting problem had an increased admission risk, while depressive symptoms were associated with lower admission risk. Patients at risk of deliberate self harm were less likely than others to be admitted, while those at risk of violence or unintentional harm to self (i.e. self neglect or reckless behaviour) had higher admission rates. Males were more likely to be admitted, as were members of the Black African (but not Black Caribbean or Black Other) ethnic group. Social problems (HoNOS ratings) and a prior history of being admitted were associated with greater likelihood of admission. Self-referral was associated with a lower admission rate, while police referral was associated with greater likelihood of admission.

Table 3 shows the results of logistic regression analysis, with the variables listed in Table 2 entered simultaneously. The patients under the care of the North Islington team remained less likely to be admitted than the others. Those described by staff as uncooperative with the process of arranging the initial assessment were also much more likely to be admitted. Further significant associations at the p = 0.05 level were with assessment outside normal working hours, assessment in the casualty department, and a moderate or severe risk of unintentional harm to self (for example through self neglect). Being younger and having a previous history of being detained under the Mental Health Act were also significantly associated with admission. The regression was also repeated without uncooperativeness, a potential proxy for hostility and risk of violence: the main change was that a highly significant association now emerged between staff-rated risk of violence and a higher risk of admission (odds ratio: 3.87, 95% confidence intervals 1.73 to 8.67, p = 0.001).

**Table 3:** Significant results of multivariate analysis for primary outcome of admission in 8 weeks

**Variable**	**Odds ratio for being admitted at 8 weeks**	**95% confidence interval**	**p-value**
**Age per increasing year**	0.97 per year (greater admission risk with younger age)	0.95 to 1.00	0.04
**Previous compulsory admission**	2.64	1.07 to 6.55	0.04
**Moderate or severe risk of unintentional self harm vs no or mild risk**	2.93	1.42 to 6.05	0.004
**Uncooperative with assessment**	10.25	4.20 to 24.97	<0.001
**Referral from Accident and Emergency department**	3.12	1.55 to 6.26	0.001
**Referral outside usual office hours**	2.34	1.11 to 4.94	0.03
**South Islington Crisis Team vs North Islington Crisis Team**	9.00	3.74 to 21.63	<0.001
**North Southwark Crisis Team vs North Islington Crisis Team**	9.79	4.20 to 22.80	<0.001

Table 4 shows results for multivariate analyses using the secondary outcomes. At 6 months the independent factors associated with hospital admission remained broadly similar to those at 8 weeks. Moderate to severe risk of harm to others was associated with admission at 6 months but not at 8 weeks. Assessment outside normal working hours and previous detention under the Mental Health Act were, however, no longer significantly related to admission at 6 months. Bed use at 8 weeks and 6 months again gave similar findings. Greater bed use was associated with treatment under the South Islington team, uncooperativeness, assessment in the casualty department, risk of unintentional harm to self and of harming others. There was no longer a significant difference in bed use between the North Southwark and North Islington CRTs by 6 months.

**Table 4:** Results of Multivariate analysis for secondary outcomes^1^

**Variable**	**Admission in 6 months after crisis**		**Bed usage in 8 weeks after crisis**		**Bed usage in 6 months after crisis**		**Compulsory admission in 6 months after crisis**	
	**OR + 95% CI**	**p-value**	**Mean difference in days between groups + 95% CI^1^**	**p-value**	**Mean difference in days between groups + 95% CI^2^**	**p-value**	**OR + 95% CI**	**p-value**

**Risk of unintentional harm to self**	2.91 (1.37 to 6.19)	0.005	7.43 (3.31 to 11.55)	<0.001	12.65 (2.48 to 22.81)	0.02	NS	NS
**Risk of harm to others**	2.46 (1.03 to 5.88)	0.04	6.08 (1.22 to 10.93)	0.01	16.31 (4.57 to 28.04)	0.007	NS	NS
**Assessed in casualty**	2.85 (1.41 to 5.73)	0.003	5.85 (1.90 to 9.80)	0.004	9.87 (0.03 to 19.71)	0.05	2.97 (1.07 to 8.29)	0.04
**Uncooperative with initial assessment**	17.70 (6.28 to 49.90)	<0.001	12.20 (7.39 to 17.00)	<0.001	19.28 (7.47 to 31.08)	0.002	13.74 (5.15 to 36.71)	<0.001
**South Islington CRT**	7.34 (3.17 to 17.00)	<0.001	6.92 (2.50 to 11.33)	0.002	17.05 (6.19 to 27.90)	0.002	NS	NS
**North Southwark CRT**	8.19 (3.58 to 18.72)	<0.001	7.39 (2.58 to 12.19)	0.003	NS	NS	NS	NS
**Age (per increasing year)**	0.97 (0.95 to 1.00)	0.04	NS	NS	NS	NS	NS	NS
**Previously sectioned**	NS	NS	NS	NS	NS	NS	5.10 (1.52 to 17.07)	0.008
**Referred by police**	NS	NS	NS	NS	NS	NS	6.58 (1.84 to 23.61)	0.004
**Black African ethnic group**	NS	NS	NS	NS	NS	NS	4.18 (1.34 to 12.97)	0.01

NS = Not significant

^1^Variables only appear in this table if they produced a significant result at p < 0.05 in at least one of the analyses.

^2^A mean difference > 0 indicates that the characteristic listed was associated with an increased number of bed days.

The set of variables associated with compulsory detention in hospital was somewhat different from that associated with admission in general. Casualty department assessment and uncooperativeness were associated with this outcome, as with admission generally, but other variables strongly associated with compulsory detention were Black African ethnic group, referral by the police and a previous history of compulsory detention.

## Discussion

### Limitations

This study incorporated data collected from three different studies using methods that were not identical, although the core set of measures was largely the same. Recruitment methods differed between the South Islington study and the other two, probably resulting in fewer exclusions of severely ill patients in South Islington than elsewhere. Thus the comparison between teams should be treated with considerable caution, even though adjustment has been made for some of the baseline differences that may have resulted from different methods of recruitment. We tested a comprehensive set of variables informed by literature or based on clinical grounds. However, some important variables may have been omitted. Recognised standardised measures were used for ratings of the severity of symptoms and social problems and of risks, but these scales are very broad and global, and more differentiated and sensitive measures of domains such as psychotic symptom severity might have yielded significant associations. The imputation of some missing values (no more than 6% of values for any variable) should be noted. It should also be noted that all findings are associations only: causality cannot necessarily be assumed.

Finally it should be mentioned that this study was carried out in inner city London which represents an area of high deprivation with a transient and cosmopolitan population. The combination of a greater severity of illness and rootlessness means that this population is not typical of the UK as a whole [30]. The high local density of large general hospitals with very busy casualty departments and the high local threshold for admission are each factors which may influence local CRT practices.

### Findings

Our findings identify factors that may limit CRTs' capacity to treat people at home. They also suggest that some factors expected in theory to limit considerably scope for home treatment may not in fact do so.

#### Service delivery

One consistent determinant was the particular CRT delivering the service. The difference in recruitment methods discussed above must be noted: even with adjustment for some baseline differences, there may be residual differences, especially between the unselected South Islington sample and the others, that may account for some of the differences found. The areas are socio-demographically fairly similar, but the possibility that differences in catchment area populations or in other aspects of the mental health service system, especially the availability in Islington of crisis houses, may account for differences. The differences found nonetheless raise questions about variations in practice and in admission thresholds between teams, and whether different implementations of the CRT model may produce substantially different results in terms of preventing admission. The content of the CRT model has not been specified in detail, so that it is perhaps best regarded as a service delivery vehicle than as a method of treatment. We need more detailed investigation of the working practices of CRTs and how these influence the effective prevention of admission. A further potentially important factor is the extent to which teams permit patients to choose to go to hospital if this is their preference. Current formulations of the model suggest that home treatment should be delivered whenever feasible and economic pressures certainly favour avoidance of admission, but the increasing emphasis on allowing service users choice conflicts with these imperatives. Participation in a randomised controlled trial may have placed staff under particular pressure to prevent admission wherever possible.

#### Context of assessment

The location of assessment also influences whether patients are admitted to hospital. Those being assessed in casualty departments were more likely to be admitted after adjustment for all other baseline variables. Possible reasons for this include casualty department time pressures, or the expectation of patients presenting to casualty departments that they will be admitted. Unmeasured differences in symptom severity are another possibility. The casualty department environment may also be one which promotes admission: clinicians are unable to assess patients' home environments and their ability to maintain order there and to cope with daily activities. A more confident appraisal of patients' coping abilities in the community and of their support networks may be possible in patients' homes, encouraging clinicians to opt for home treatment. The environment of the casualty department may also influence patients' behaviour. Our finding regarding casualty department assessments suggests that assessment at home is desirable wherever possible; even following initial attendance to the casualty department. Patients assessed outside usual office hours were also more likely to be admitted to hospital within 8 weeks of the crisis. This might be due to undetected differences in psychopathology, as assessments may take place out of hours because of greater perceived urgency. Lower staffing levels, more junior staff and lack of other resources are other possible explanations.

#### Patient characteristics and past history

With regards to the characteristics of the patients, the strongest and most consistent finding was that patients who had not been cooperative with the process of arranging the initial assessment were much more likely to be admitted: this association was much stronger than with any clinical variables, suggesting that patients' willingness to engage with services is likely to be the most important single determinant of whether home treatment is feasible. Risk of unintentional self-harm was also a risk factor for admission: self neglect may be more readily manageable with 24-hour staff in hospital than in a home setting. Risk to others also increased risk of admission, reflecting greater caution in managing patients who may be a danger to others or difficulties with home management because of perceived risk to staff. The association between past history of being sectioned under the mental health act and admission suggests that past patterns of crisis management may be difficult to change, although no statistically significant independent association was found between admission in the past 2 years and admission following the crisis.

#### Negative findings

Comparing our findings with previous studies, there were also some noteworthy negatives. Diagnostic variables did not contribute to likelihood of admission in the final model, and patients with manic presentations, current psychotic symptoms, comorbid substance misuse and personality disorders were all managed at home in substantial numbers. Living alone was also unrelated to risk of admission: while home treatment may be easier for people who have a support network, the impact of introducing a CRT may be greater for a patient who has little other support. Ethnic group also had no clear association with risk of admission when adjustment was made for other variables. Numbers were not large enough to draw any definitive conclusions, but this provides at least some preliminary encouragement that the CRT model may be applicable to people from a range of backgrounds. However, the higher rate of admission for the Black African group, significant on univariate analysis, together with this group's increased risk of compulsory admission, suggests a need to pay particular attention to the needs of this group in future planning of crisis services.

#### Compulsory admissions

Factors associated with compulsory detention were somewhat different from the others. This was the one outcome not associated with which team treated the patient, suggesting that variations between teams related more to voluntary than compulsory admission. It was also a variable on which ethnic group had a large impact: while in past studies Black Caribbean patients have appeared at greater risk of coercive treatment, we found no evidence of this, but did find an association between Black African group and risk of being compulsorily detained. Which CRT was involved did not influence likelihood of compulsory admission, perhaps not surprisingly given that the Islington studies suggested that overall the impact on admissions was on voluntary rather than compulsorily detained patients [7,8].

### Future service delivery and research

In future research regarding CRTs, greater investigation of the mechanisms underlying these differences in admission rate would be of interest. Analysis of the process of decision making in the early management of crises may enhance understanding of the variations described. Some of the risk factors for admission are potentially amenable to change: for example, avoiding initial assessments in the casualty department might prevent some admissions. A greater understanding is also desirable of patients' views and the factors that make some of them unwilling to cooperate with assessment. In our sample, only a small number of patients were admitted after a week or more of community management, so that there was relatively little scope for examining the characteristics of this group for whom a substantial period of home treatment appears to have failed to prevent admission: focusing on them may be of interest in future investigations. With regard to implications for in-patient wards, this study suggests that the group admitted despite the availability of home treatment is likely to be a relatively challenging one, with high representation of young patients who are unwilling to cooperate with care and pose risks of self neglect or harm to others. In-patient staff is thus likely to require substantial support and training, and good staffing levels in order to manage these groups. The effects on in-patient services of the introduction of intensive home treatment have yet to be examined. Finally, identifying the characteristics of patients who are not successfully managed by home treatment allows consideration of other alternatives to admission that may meet their needs better. For example, residential alternatives to admission, such as crisis houses, may be better placed than CRTs to manage the needs of those who neglect themselves.

## Conclusion

The type of CRT support, the context of assessment and patient characteristics are all factors that appear to influence whether a patient in crisis is successfully treated in the community. Identifying these features has implications for future development of CRT and inpatient provision and of other alternatives to admission.

## Competing interests

The author(s) declare that they have no competing interests.

## Authors' contributions

MC +SJ had the concept and design for the paper. They conducted the main analysis and interpretation of data and drafted the manuscript.

JB, AS, IW, GT, FN, SP, JH, NM and PB all made substantial contributions to the design of the paper, interpretation of data and revised for important intellectual content.

In addition FN and AS made significant contributions to data acquisition and IW made a substantial contribution to data analysis.

All authors have given final approval to drafting the manuscript.

## Pre-publication history

The pre-publication history for this paper can be accessed here:


